# Circulating tumor DNA for surveillance in high-risk melanoma patients: a study protocol

**DOI:** 10.2340/1651-226X.2025.42515

**Published:** 2025-02-10

**Authors:** Magnús P.B. Obinah, Sarah A. Al-Halafi, Karin Dreisig, Tim S. Poulsen, Christoffer Johansen, Thomas Litman, Stig E. Bojesen, Estrid Høgdall, Annette H. Chakera, Lisbet R. Hölmich

**Affiliations:** a Department of Plastic Surgery, Copenhagen University Hospital – Herlev and Gentofte, Copenhagen Denmark; b Department of Clinical Biochemistry, Copenhagen University Hospital – Herlev and Gentofte, Copenhagen Denmark; c Molecular Unit, Department of Pathology, Copenhagen University Hospital – Herlev and Gentofte, Copenhagen Denmark; d Center for Cancer Late Effect Research CASTLE, Department of Oncology, Copenhagen University Hospital – Rigshospitalet, Copenhagen, Denmark; e Department of Immunology and Microbiology, Faculty of Health and Medical Sciences, University of Copenhagen, Copenhagen, Denmark; f Department of Clinical Medicine, Faculty of Health and Medical Sciences, University of Copenhagen, Copenhagen, Denmark; g Agata Private Hospital, Copenhagen, Denmark

**Keywords:** Liquid biopsy, biomarker, personalized surveillance, molecular monitoring

## Abstract

**Background and purpose:**

Melanoma is one of the deadliest skin cancers and challenges clinicians worldwide due to rising incidence, potential aggressiveness, and propensity for metastasis, necessitating comprehensive follow-up programs after primary treatment.

Circulating tumor DNA (ctDNA) is a promising biomarker that may indicate disease progression earlier than traditional surveillance methods, including 18F-FDG PET-CT, ultrasound, and clinical examination. This study examines ctDNA detection in blood as a minimally invasive method for early identification of progression following primary treatment of melanoma. The aim is to overcome the limitations of current methods, potentially improving prognosis and survival.

**Patients/material and methods:**

Patients with high risk of recurrence following primary treatment of melanoma are offered inclusion. Blood sampling is performed at each follow-up visit. In case of recurrence, patient-specific mutations are identified through next-generation sequencing (NGS) of formalin and paraffin embedded tissue from diagnostic routine. Detection of mutation-specific ctDNA is performed on blood using digital droplet polymerase chain reaction (ddPCR) or NGS. This allows determination of the value and sensitivity of ctDNA for early detection of recurrence.

**Results and Interpretation:**

For validation purposes, we conducted a small pilot study using blood samples from 10 patients who had experienced recurrence and had a clinically confirmed *BRAF V600E* mutation. Detection of *BRAF V600E* ctDNA using ddPCR varied from 0/5 (0%) in DNA harvested from 4 mL plasma, to 3/5 (60%) in DNA from 8 mL of plasma. These results show promise and highlight the importance of high sensitivity and sampling volumes to ensure accurate detection of low levels of ctDNA.

## Introduction

Melanoma, characterized by increasing incidence, aggressiveness, and propensity for both early and late metastasis, requires comprehensive follow-up after primary treatment. Advances in treatment outcomes for recurrence and metastatic disease, have made early detection of progression increasingly important [1], yet current surveillance methods have limitations. Whole-body 18F-FDG PET-CT, the current gold standard for detecting internal metastases, is limited to detecting lesions larger than 4–6 mm, exposes patients to radiation, has high costs and is prone to false-positive results [2–4]. Current biomarkers, lactate dehydrogenase (LDH) and serum S100B, are both non-specific and elevated by benign and malignant conditions [5, 6].

Circulating Tumor DNA (ctDNA), a subset of normal cell-free DNA (cfDNA), has recently emerged as a promising biomarker for monitoring cancer progression [7]. Consisting of predominantly short DNA fragments, released from tumor cells due to apoptosis, necrosis, or active secretion, ctDNA may be detected in various biofluids [8–10]. For ctDNA detection in blood, plasma is a preferable to serum, which contains high amounts of normal wild-type cfDNA, effectively lowering the detectable ctDNA fraction [11]. Detection of ctDNA can be challenging, due to its brief half-life, low abundance and high variability based on tumor burden, location and biological features such as histological parameters, vascularization, proliferation, and apoptosis rates [12–15], requiring reliable detection of mutant allele frequencies as low as ≤ 0.1% [16, 17]. Current methods, including digital droplet polymerase chain reaction (ddPCR) and targeted deep next generation sequencing (NGS), can detect low ctDNA concentrations. A tumor-informed approach – where analysis of tumor-tissue is used to identify patient-specific mutations, for targeted detection of mutation-specific ctDNA in biofluids – can further enhance the specificity and sensitivity of detection at lower concentrations, and a limit of detection (LoD) as low as 0.0005% has been reported using ddPCR for specific mutations [17–19].

Melanoma is characterized by a high tumor mutation burden, making it a prime candidate for ctDNA applications [20]. Approximately 83% of melanomas exhibit either a *BRAF* (63%) or *NRAS* (26%) driver mutation, typically mutually exclusive [21, 22]. ctDNA measurement in melanoma has shown promise for various applications, including prediction of survival and response to therapy, monitoring tumor burden and activity, and detection of minimal residual disease [23–26]. Preliminary data indicate that ctDNA may also detect disease progression, possibly earlier than current imaging techniques [27]. This requires further validation however, and future clinical implementation based on such proof-of-principle studies will require standardization of associated clinical and analytical variables [28].

This study aims to investigate the role of ctDNA in the early detection of melanoma progression. By comparing the sensitivity of ctDNA with current methods in high-risk patients with known recurrence, we aim to determine its value for early detection in personalized surveillance [29]. Our hypothesis is that detection of ctDNA in plasma will prove a minimally invasive, repeatable, and affordable method for reliable detection of progression in high-risk melanoma patients, potentially earlier than PET-CT.

## Patients/materials and methods

This prospective, single-institution study will recruit patients above 18 years of age, who attend follow-up after treatment for primary melanoma stages IIB-III or resected stage IV, during an inclusion period, from July 1^st^, 2019, to October 6^th^, 2022, at Copenhagen University Hospital – Herlev and Gentofte, Denmark. Exclusion criteria are pregnancy and previous disseminated melanoma. We expect to enrol approximately 450 patients during the inclusion period, of which 118 are expected to progress within 2-years of enrolment.

Enrolled patients who provide written informed consent will be followed for the duration of their follow-up program, expected to be 5-years or until termination of clinical follow-up for any reason, with blood sampling at each follow-up visit. The total study-period will be 2019 – 2027. The surveillance program for these patients entails clinical examinations every 3 months for 2 years, and hereafter every 6 months for 3 years, with PET-CT scans at baseline and after 6, 12, 24, and 36 months, and ultrasound of relevant lymph node basins at other times.

Blood samples (2 × 9 mL ethylenediaminetetraacetic acid (EDTA) tubes) will be centrifuged at 3,000 g for 6 min to recover 8 mL of plasma within 3 h of sampling, for storage in a biobank at −80°C. Lactate dehydrogenase (LDH), high-sensitivity C-reactive protein (HS-CRP), and white blood cell differential count (WBC diff.) will also be analysed at each sampling.

In cases of confirmed progression, biopsied or biobank tumor tissue will be analysed using the Oncomine Tumor Mutation Load Assay NGS panel (Thermo Fisher Scientific, Waltham, Massachusetts, U.S.), to determine the specific patient mutational profile. Plasma DNA will be extracted from samples closest to the initial suspicion of progression after secondary centrifugation at 3,000 g for 6 min, using the Qiasymphony DSP Circulating DNA kit (Qiagen, Hilden, Germany), and analyzed for patient-specific ctDNA using either the Bio-Rad QX200 Droplet Digital PCR System (Bio-Rad Laboratories, Hercules, California, United States) or the Oncomine Tumor Mutation Load Assay NGS panel (Thermo Fisher Scientific, Waltham, Massachusetts, U.S.), pending ddPCR assay availability and validity.

Primary outcomes of this study include diagnostic sensitivity of ctDNA for detection of progression, (defined as local recurrence or any kind of metastasis). Secondary outcomes include time from detectable ctDNA to clinical or radiological progression, and associations with other biomarkers, including LDH, WBC differential, and HS-CRP.

An interim analysis will be performed ultimo 2024, including all patients with confirmed recurrence by this time, expected to be published in 2025. A final analysis will include all recurrences confirmed by 2027.

This protocol study follows the Standard Protocol Items: Recommendations for Interventional Trials (SPIRIT) guidelines [30]. The SPIRIT checklist and figure are provided as supplementary information. This study is approved by the Danish Research Ethics Committees (H-18008586) and registered on ClinicalTrials.gov (NCT06246227).

## Results

A pilot study was initiated in January 2022 to validate the method, in which we identified through chart-review, a total of 10 included patients who (1) had experienced progression during the study period, (2) had a previously confirmed *BRAF V600E* tissue mutation and (3) had a blood sample within a specific number of days from the date of suspicion, for what was later confirmed as progression (−95 to +14 days), where the likelihood of detectable ctDNA would be greatest and detection would still provide clinical value comparable to current methods.

NGS analysis of biobank tissue confirmed *BRAF V600E* mutation in nine patients, while for one patient, no tissue was available for confirmation analysis.

Early in the study, the sampling volume was increased from 4 to 8 mL of EDTA plasma to ensure sufficient DNA for detecting ctDNA at low concentrations. Among the 10 included samples, five were collected before this change and contained DNA extracted from 4 mL of plasma, while five collected after contained DNA extracted from 8 mL of plasma. All samples were concentrated to 60 μL of purified DNA, of which 5 μL was used as input for ddPCR-based detection of *BRAF V600E* ctDNA. Positive droplets were counted to calculate the copy number of mutated and wild type cfDNA in plasma. Samples were called positive if the mutated copy concentration exceeded 3.6 copies/mL for 8 mL plasma or 7.2 copies/mL for 4 mL plasma, based on internally established and clinically validated threshold limits.

In DNA samples derived from 4 mL of plasma, *BRAF V600E* ctDNA was detected in 0/5 (0%). In DNA samples derived from 8 mL of plasma, *BRAF V600E* ctDNA was detected in 3/5 (60%). Detected ctDNA levels ranged from 8 to 2,301 copies/mL (Table 1).

**Table 1:** Detection of BRAF V600E ctDNA using ddPCR (BioRad QX200).

ID	Protocol	BRAF V600E	Mutant Allele Frequency (MAF)	Suspicion to Sampling (Days)	Type of Progression
10	8 mL	2,301 copies/mL	0.2345	−5	Multifocal
9	8 mL	8 copies/mL	0.0036	−11	Multifocal
8	8 mL	ND	ND	−95	Multifocal
7	8 mL	19 copies/mL	0.0085	2	Multifocal
6	8 mL	ND	ND	−47	Multifocal
5	4 mL	ND	ND	4	Locoregional
4	4 mL	ND	ND	14	Multifocal
3	4 mL	ND	ND	−85	Multifocal
2	4 mL	ND	ND	10	Locoregional
1	4 mL	ND	ND	−50	Multifocal

ND: Not Detected; Locoregional: local recurrence, in-transit and/or regional lymph node metastases; Multifocal: metastases in more than 2 remote (non-loco-regional) locations relative to the site of the primary tumor (e.g. liver and lung, or lung and mediastinal lymph nodes).

## Discussion

This study investigates ctDNA-guided surveillance for high-risk melanoma patients following primary treatment. A minimally invasive method, ctDNA has the potential to enhance both sensitivity and specificity through molecular targeting, while improving affordability and patient-safety compared to current methods. The strengths of our study include its prospective design, standardized sampling protocols, and the use of advanced standardized molecular techniques like ddPCR and NGS.

The tumor-informed approach of identifying tissue mutations prior to ctDNA detection of identified (patient-specific) mutations in blood samples, allows broader applicability and selecting the optimal detection-method for each specific mutation based on availability and reliability. This method provides inherent advantages and should provide higher specificity than FDG-PET’s detection of non-specific uptake patterns. While technical artifacts in calling mutations and rare phenomena such as clonal hematopoiesis of indeterminate potential (CHIP) could theoretically lead to false positives, these can be mitigated through stringent analysis protocols and further steps such as analyzing buffy coat to exclude CHIP-related variants.

Limitations include the single-center design and potential selection bias in the high-risk population studied. By analyzing only samples from patients with confirmed progression, we can establish the overall detection capability of the proposed method but are unable to directly assess false-positive rates. This choice was made to (1) establish analytical validity before broader implementation, (2) avoid potential unnecessary interventions and patient anxiety, and (3) manage cost-effectiveness by focusing on confirming known cases of progression. All our samples are stored at −80°C following standardized procedures, enabling potential future analysis if additional resources or methods become available.

Our pilot data highlight the importance of securing sufficient sampling volumes to ensure detectable amounts of ctDNA. Our detection rates varied from 0% in DNA harvested from 4 mL of plasma to 60% in DNA harvested from 8 mL of plasma. The increase in sampling volume was decided before the pilot study, based on advice from other research groups, to reflect our focus on early detection of progression, where ctDNA concentrations are expected to be very low. For other ctDNA applications, for example, monitoring of treatment response where higher mutant allele frequencies are expected, 4 mL of plasma may suffice.

While two of seven ctDNA negative patients had loco-regional recurrence, we found no other variations between patients with detectable and undetectable ctDNA. However, our ability to correlate ctDNA detection with disease burden was limited, as we did not use a standardized method for classifying tumor burden (e.g. RECIST criteria). This limitation was deemed acceptable, given the pilot study’s primary aim of validating our hypothesis and method. The main study will include a standardized classification of tumor burden to enable such analysis.

Of note, other blood-based methods such as biomarkers including S100B and LDH, require smaller sampling volumes but lack specificity, and emerging methods such as methylation analysis, fragment-length assessment, and cell-free RNA analysis, while promising, remain complex and non-standardized.

In the pilot study, ddPCR was used to detect *BRAF V600E* ctDNA as our laboratory has a clinically validated assay targeting this mutation. The choice of a molecular method requires careful consideration – while ddPCR offers high sensitivity for known mutations, a broader NGS approach may detect a wider range of known and unknown alterations, but in turn require more complex analysis and interpretation by highly specialized personnel.

Other factors such as surgery, tumor burden, and location must also be considered, as they can influence the absolute and relative ctDNA quantities in sampled blood.

The clinical value of a method for recurrence detection will ultimately depend on analytical sensitivity, with a highly sensitive assay potentially capable of detecting minor disease progression, enabling complete surgical resection of metastases, potentially lengthening disease-free periods. While our study design does not allow for direct calculation of specificity or predictive values, we will discuss the implications of our findings on these parameters based on test characteristics and results.

This study aims to evaluate the clinical utility of ctDNA-based surveillance strategies for the care of melanoma patients, in two interconnected areas: (1) The detection capability and temporal relationship of ctDNA compared with current imaging methods at time of progression and (2) The diagnostic sensitivity of ctDNA for individual mutational profiles, using standardized blood sampling and molecular analysis techniques.

These findings may provide a foundation for further exploring the role of ctDNA in personalized medicine, such as determining the clinical benefits of ctDNA-based surveillance for specific subgroups stratified by mutational profiles and progression patterns. In such future prospective studies, analyzing samples in real-time would be valuable to determine true false-positive rates.

## Supplementary Material

Circulating tumor DNA for surveillance in high-risk melanoma patients: a study protocol

## Data Availability

Due to patient confidentiality requirements, access to the final dataset will be restricted to active clinical members of the study group. Qualified researchers may request access through the corresponding author, subject to appropriate data sharing agreements and ethical approvals.

## Trial registration

This study is registered on ClinicalTrials.gov (NCT06246227).
